# Enhancement of Commercial Antifungal Agents by Kojic Acid

**DOI:** 10.3390/ijms131113867

**Published:** 2012-10-26

**Authors:** Jong H. Kim, Perng-Kuang Chang, Kathleen L. Chan, Natália C. G. Faria, Noreen Mahoney, Young K. Kim, Maria de L. Martins, Bruce C. Campbell

**Affiliations:** 1 Plant Mycotoxin Research Unit, Western Regional Research Center, USDA-ARS, 800 Buchanan St., Albany, CA 94710, USA; E-Mails: kathy.chan@ars.usda.gov (K.L.C.); noreen.mahoney@ars.usda.gov (N.M.); ykkim@kookmin.ac.kr (Y.K.K.); bruce.campbell@ars.usda.gov (B.C.C.); 2 Food and Feed Safety Research Unit, Southern Regional Research Center, USDA-ARS, 1100 Robert E. Lee Blvd., New Orleans, LA 70124, USA; E-Mail: perngkuang.chang@ars.usda.gov; 3 Instituto de Higiene e Medicina Tropical/CREM, Universidade Nova de Lisboa, Portugal; E-Mails: natalia.faria@insa.min-saude.pt (N.C.G.F.); luzmartins@ihmt.unl.pt (M.L.M.); 4 Department of Forest Products and Biotechnology, College of Forest Sciences, Kookmin University, Seoul 136-702, Korea

**Keywords:** Kojic acid, hydrogen peroxide, amphotericin B, strobilurin, chemosensitization

## Abstract

Natural compounds that pose no significant medical or environmental side effects are potential sources of antifungal agents, either in their nascent form or as structural backbones for more effective derivatives. Kojic acid (KA) is one such compound. It is a natural by-product of fungal fermentation commonly employed by food and cosmetic industries. We show that KA greatly lowers minimum inhibitory (MIC) or fungicidal (MFC) concentrations of commercial medicinal and agricultural antifungal agents, amphotericin B (AMB) and strobilurin, respectively, against pathogenic yeasts and filamentous fungi. Assays using two mitogen-activated protein kinase (MAPK) mutants, *i.e*., *sakA*Δ, *mpkC*Δ, of *Aspergillus fumigatus*, an agent for human invasive aspergillosis, with hydrogen peroxide (H_2_O_2_) or AMB indicate such chemosensitizing activity of KA is most conceivably through disruption of fungal antioxidation systems. KA could be developed as a chemosensitizer to enhance efficacy of certain conventional antifungal drugs or fungicides.

## 1. Introduction

**Figure 1:**
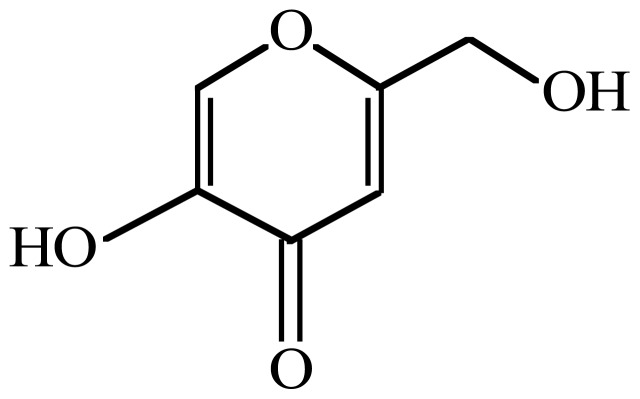
Structure of kojic acid (KA).

Kojic acid (KA, Figure 1) is a natural pyrone produced by certain filamentous fungi, mainly species of *Aspergillus* and *Penicillium*. It is a common by-product in the fermentation of soy sauce, sake and rice wine, and is widely used as a food additive to prevent oxidative browning, or in cosmetics as a depigmenting agent [1–3]. Genes involved in KA biosynthesis were recently identified [4,5]. Cellular immunity is enhanced by KA through stimulating phagocytosis and generation of reactive oxygen species (ROS) in macrophages, and potentiation of phytohemagglutinin-based proliferation of lymphocytes [6,7]. KA is fungistatic against the pathogenic yeast, *Cryptococcus neoformans*, by inhibiting melanin production required for infectivity [8]. Derivatives of KA also have antimicrobial activity against a variety of other fungi and bacteria [9], showing its potential as a polyfunctional backbone for new antimicrobial agents [10].

Among *Aspergillus* species, *A. flavus*, *A. parasiticus* and *A. oryzae* are the main producers of KA [11]. *A. oryzae* is used widely in the food industry. However, *A. flavus* and *A. parasiticus* are opportunistic pathogens of various crops, and a concern since they produce carcinogenic aflatoxins that can contaminate food. *A. flavus* is also an agent for human invasive aspergillosis (IA). Of note, the chief agent of IA, *A. fumigatus*, and a third IA agent, *A. terreus*, do not produce KA [12–14].

Co-application of certain types of compounds can enhance efficacy of conventional antimicrobial agents through a process termed “chemosensitization.” With regard to microbial pathogens, a chemosensitizer functions by debilitating the ability of a pathogen to completely activate a defense mechanism to an antimicrobial agent [15,16]. We investigated if KA, as a chemosensitizer, could improve activity of commercial antifungal agents against pathogenic strains of *Aspergillus* and yeasts (See Table 1). We tested this chemosensitizing potential by co-applying KA with hydrogen peroxide (H_2_O_2_) to mimic host ROS, and with a commercial antimycotic, amphotericin B (AMB) and agricultural fungicides, fludioxonil (FLUD) and strobilurin (kresoxim methyl (Kre-Me)).

## 2. Results and Discussion

### 2.1. Enhanced Antimycotic Activity of H_2_O_2_ by KA against Filamentous Fungi

#### 2.1.1. Agar Plate Bioassay: Filamentous Fungi

We initially tested KA (5 mM) and H_2_O_2_ (3, 4, 5, 6 mM) on filamentous fungal growth, comparing colony diameter to controls in agar bioassays (See Experimental Section). Three strains of *A. fumigatus* (wild type strain, AF293, and two deletion mutants for oxidative/osmotic stress responsive mitogen-activated protein kinase (MAPK), *sakA*Δ and *mpkC*Δ) [17,18], three clinical strains of *A. terreus* (UAB-673, −680 and −698), and one wild type strain, each, of *A. flavus* (NRRL3357) and *A. parasiticus* (NRRL5862), were tested. Fungi were cultured at 35 °C, except *A. parasiticus* at 28 °C, on potato dextrose agar (PDA).

**Figure 2:**
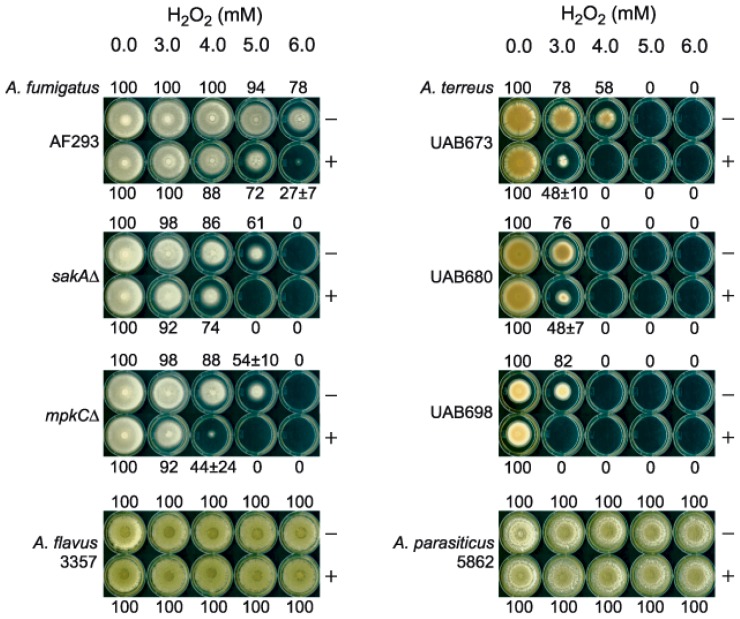
Agar bioassay showing antifungal chemosensitization of kojic acid (KA) with H_2_O_2_ tested against *Aspergillus* strains. Numbers (0–100) indicate percent (%) radial growth compared to non-treated control (100%; no H_2_O_2_ and no KA). (−), w/o KA; (+), w/KA (5 mM).

Results showed (Figure 2): (1) KA (at 5 mM) did not affect growth of any strain; (2) H_2_O_2_ (up to 6 mM) alone or with KA had no effect on *A. flavus* or *A. parasiticus*; (3) H_2_O_2_ alone or with KA inhibited growth of all strains of *A. fumigatus* and *A. terreus*. Strain sensitivity to KA + H_2_O_2_ varied as follows (in decreasing order): *A. terreus* UAB698 > strains 680 = 673 > *A. fumigatus mpkC*Δ = *sakA*Δ > AF293 > *A. flavus* = *A. parasiticus*. Therefore, KA + H_2_O_2_ treatments inhibited growth much more significantly in strains that do not produce KA (*i.e*., *A. fumigatus*, *A. terreus*).

#### 2.1.2. Microtiter Plate (microdilution) Bioassay: Filamentous Fungi

Based on results of the agar bioassay (shown above), antifungal interactions between KA and H_2_O_2_ were assessed further for only the *A. fumigatus* and *A. terreus* strains using triplicate, microtiter-plate checkerboard bioassays (Clinical Laboratory Standards Institute (CLSI) M38-A) [20] with concentration ranges of KA, 0.2–12.8 mM, and H_2_O_2_, 0.0625–16 mM (See Experimental Section).

Minimum inhibitory concentrations (MICs), lowest concentration of agent(s) showing no visible fungal growth, were assessed after 48 h. Minimum fungicidal concentrations (MFCs), lowest concentration of agents showing ≥99.9% fungal death, were determined (following completion of MIC assays) wherein entire volumes of microtiter wells (200 μL) were spread onto individual PDA plates, and cultured for another 48 h. Compound interactions, Fractional Inhibitory Concentration Indices (FICI) and Fractional Fungicidal Concentration Indices (FFCI) were calculated, as follows: FICI or FFCI = (MIC or MFC of compound A in combination with compound B/MIC or MFC of compound A, alone) + (MIC or MFC of compound B in combination with compound A/MIC or MFC of compound B, alone). Interactions were defined as: “synergistic” (FICI or FFCI ≤ 0.5) or “indifferent” (FICI or FFCI > 0.5–4) [21].

**Figure 3:**
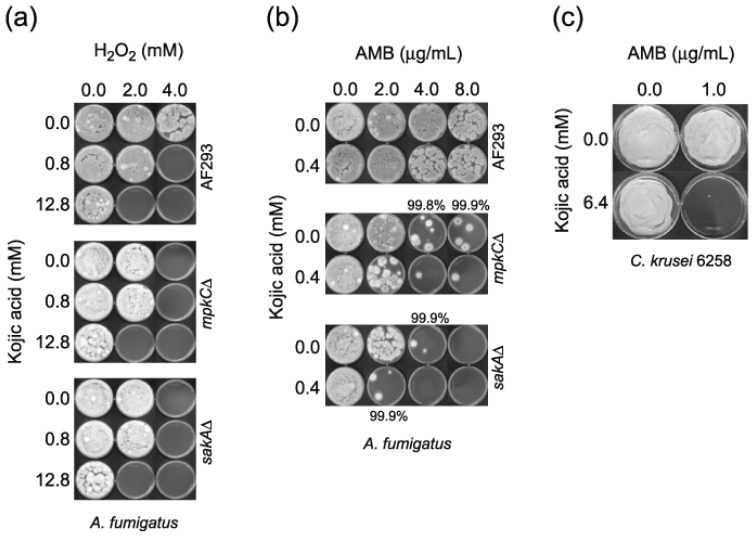
(**a**) MFC determination of *A. fumigatus* strains (AF293, *sakA*Δ, *mpkC*Δ) with the treatment of kojic acid (KA) + H_2_O_2_. (**b**) MFC determination in *A. fumigatus sakA*Δ strain with the treatment of KA + AMB. Results indicated that *A. fumigatus* AF293 and *mpkC*Δ strains needed higher concentration of KA or AMB to achieve ≥99.9% cell death compared to *sakA*Δ. (**c**) MFC determination in *Candida krusei* ATCC6258 with the treatment of KA + AMB.

Synergistic FICIs and FFCIs between KA and H_2_O_2_ only occurred in AF293. Despite the absence of calculated “synergism” as depicted by “indifferent” interactions (by definition) (Table 2), there was enhanced antifungal activity (*i.e*., chemosensitization) in the remaining *A. fumigatus* and *A. terreus* strains. This enhancement was indicated by lower MICs and MFCs for either or both KA and H_2_O_2_ when co-applied. Also, the *A. fumigatus* MAPK mutants had half the MICs and MFCs of AF293 (Table 2; Figure 3a), suggesting that, in the wild type fungi, MAPKs in the oxidative/osmotic stress responsive pathway play protective roles against the antimycotic activity of KA + H_2_O_2_.

### 2.2. Enhanced Antimycotic Activity of AMB with KA in Filamentous Fungi and Yeasts

AMB is an antimycotic drug against filamentous or yeast pathogens. However, AMB can be associated with significant side effects resulting in nephrosis and other tissue-damage in invasive pulmonary aspergillosis [23]. Therefore, we reasoned that use of chemosensitizing agents from natural sources could enhance the effectiveness of AMB, while lowering toxicity of this polyene drug to human cells. The main mode of action of AMB is disruption of the fungal plasma membrane, resulting in ion leakage. However, AMB also induces oxidative damage [24–27] by stimulating ROS production [28]. Since KA contributed to oxidative stress when combined with H_2_O_2_ in *Aspergillus* (See Table 2), we surmised it might also enhance AMB activity.

#### 2.2.1. Microtiter Plate (microdilution) Bioassay: Filamentous Fungi

Checkerboard assays of KA (0.2–12.8 mM) and AMB (0.125–32 μg/mL) (See Experimental Section) were initially used to assess antifungal interactions against the *Aspergillus* strains, by using CLSI M38-A protocol [20]. In assays of the *Aspergillus* strains, co-application of KA increased AMB activity only in strains of *A. fumigatus*, where FICIs and FFCIs were synergistic in the *A. fumigatus* MAPK mutant strains (Table 3; Figure 3b).

#### 2.2.2. Microtiter Plate (microdilution) Bioassay: Yeasts

Checkerboard assays of the yeast strains employed methods outlined in the European Committee on Antimicrobial Susceptibility Testing (EUCAST)] [29]. According to these methods, MICs were determined at 24 h for *Candida* and *Saccharomyces*, and at 48 h for *Cryptococcus*. Following MIC determinations, MFCs were determined on Yeast Peptone Dextrose (YPD) agar, where cells were cultured for an additional 48 h for *Candida*/*Saccharomyces* or 72 h for *Cryptococcus*, respectively.

Among the *Candida* and *Cryptococcus* strains tested, KA enhanced AMB activity in *C. albicans* CAN276, *C. krusei* ATCC6258, *C. neoformans* CN24 (Table 3). Synergism of KA + AMB was observed in *C. krusei* ATCC6258 and *C. neoformans* strains (Table 3; Figure 3c).

In parallel checkerboard assays of *S. cerevisiae*, the wild type and two MAPK cell wall integrity mutant strains, *i.e*., *slt2*Δ (MAPK deletion; cell wall integrity pathway) and *bck1*Δ (MAPK kinase kinase deletion; cell wall integrity pathway) were included. We tried to determine whether the MAPK system for cell wall integrity plays a protective role against the antimycotic activity of KA + AMB. These mutants previously showed hypersensitivity to certain environmental stresses [30,31]. However, the mutants were not more sensitive than the wild type to co-application of either compound (Table 4), indicating Slt2p and Bck1p (*viz.*, cell wall integrity pathway) do not participate in yeast cell homeostasis under KA + AMB treatment.

### 2.3. No Enhancement of Antimycotic Activity of H_2_O_2_ with KA in Yeasts

KA (5 mM) and H_2_O_2_ (2 and 3 mM) co-application was tested against yeast in agar bioassays, including five clinical strains of *Candida*, one of *C. neoformans* and non-pathogenic, *S. cerevisiae*. Yeast cells (1 × 10^6^) were serially diluted (10-fold), spotted onto Synthetic Glucose (SG) agar incorporated with KA and/or H_2_O_2_, and incubated at 30 °C, *S. cerevisiae*, or 35 °C, *Candida*/*Cryptococcus* (See [32] for methods). These assays revealed no effect (data not shown) and hence, checkerboard assays to determine MICs, FICIs, *etc.*, were not performed.

The results of all chemosensitization tests (*i.e*., KA + H_2_O_2_ or AMB in filamentous and yeast strains) are summarized in Table 5.

### 2.4. Enhanced Antimycotic Activity of Strobilurin with KA in *A. fumigatus*

**Figure 4:**
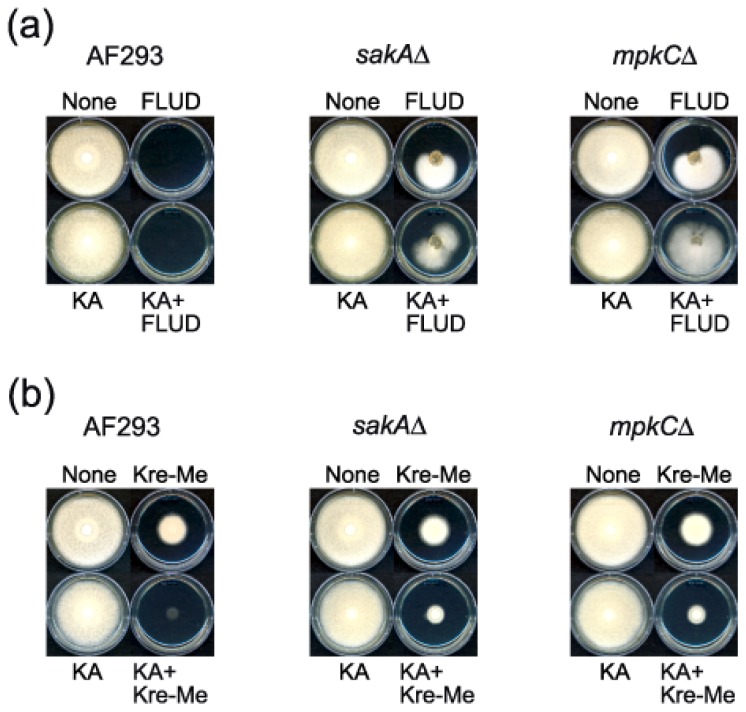
(**a**) Agar bioassay showing co-application of kojic acid (KA) could not overcome the tolerance of *Aspergillus fumigatus sakA*Δ and *mpkC*Δ mutants to fludioxonil (FLUD). None, no treatment control; FLUD 50 μM; KA 30 mM. (**b**) Agar bioassay showing co-application of KA enhanced the antifungal activity of strobilurin (Kre-Me) in *A. fumigatus* strains. None, no treatment control; Kre-Me 25 μM; KA 25 mM.

We also tested combinations of KA with agricultural fungicides, fludioxonil (FLUD) or Kre-Me (strobilurin), fungicides that target different components of the oxidative stress response system [33,34], by using *A. fumigatus* wild type and MAPK (*sakA*Δ, *mpkC*Δ) mutants. Certain fungi with mutations in genes involved in signal transduction of stress response, e.g., MAPK signaling pathway, can escape toxicity of the commercial fungicide FLUD [34]. In a prior study we found redox-active benzo derivatives co-applied with either of these fungicides reduced effective dosages and prevented tolerance of *A. fumigatus sakA*Δ and *mpkC*Δ mutants to FLUD [35]. However, in our present study, co-application of KA with FLUD did not overcome tolerance of these mutants to this fungicide (Figure 4a).

In a parallel study, we tested combinations of KA with Kre-Me. Kre-Me is an inhibitor of complex III of the mitochondrial respiratory chain (MRC), the key route system for cellular energy (ATP) production [36]. Moreover, disruption of complex III of the MRC results in an abnormal release of electrons that additionally cause cellular oxidative stress [37]. Therefore, antioxidant enzymes play important roles in protecting cells from oxidative damage triggered by MRC inhibitors. KA improved antimycotic activity of Kre-Me against all *A. fumigatus* strains (Figure 4b), where *A. fumigatus sakA*Δ and *mpkC*Δ mutants showed relatively higher tolerance to Kre-Me than the wild type (AF293). Thus, results indicated that the chemosensitizing mechanism of KA might not involve glutathione/superoxide dismutase-based oxidative stress response, differing from redox-active benzo derivatives [35]. We speculated that, in addition to inhibiting ATP production, co-application of KA and Kre-Me might involve responses of other types of antioxidant enzymes/systems. Comprehensive chemosensitization tests using KA with additional strobilurins are currently underway in various filamentous fungi, including *Aspergillus*, *Penicillium*, *Acremonium*, *Scedosporium*, and others (Note: There was no chemosensitization effect of KA with any azole drug, such as fluconazole, ketoconazole, itraconazole, in *Aspergillus* or yeasts (data not shown)).

## 3. Experimental Section

### 3.1. Fungal Strains and Culture Conditions

*Aspergillus* strains (See Table 1) were grown at 35 °C on potato dextrose agar (PDA; Sigma, St. Louis, MO, USA), except *A. parasiticus*, which was grown at 28 °C on PDA. Yeast strains (*Candida albicans*, *C. krusei*, *C. tropicalis*, *Cryptococcus neoformans*, *Saccharomyces cerevisiae*; See Table 1) were cultured on Synthetic Glucose (SG; Yeast nitrogen base without amino acids 0.67%, glucose 2% with appropriate supplements: uracil 0.02 mg/mL, amino acids 0.03 mg/mL) or Yeast Peptone Dextrose (YPD; Bacto yeast extract 1%, Bacto peptone 2%, glucose 2%) medium at 35 °C for yeast pathogens (*Candida*, *Cryptococcus*) or 30 °C for *S. cerevisiae*, respectively.

### 3.2. Chemicals

Antifungal chemosensitizing agent (kojic acid (KA)), antifungal drugs (amphotericin B (AMB), fluconazole, ketoconazole, itraconazole), strobilurin (kresoxim methyl (Kre-Me)) and oxidizing agent (hydrogen peroxide (H_2_O_2_)) were procured from Sigma Co. (St. Louis, MO, USA). Each compound was dissolved in dimethyl sulfoxide (DMSO; absolute DMSO amount: <1% in media), except H_2_O_2_, which was dissolved in water, before incorporation into culture media. In all tests, control plates (*i.e*., “No treatment”) contained DMSO at levels equivalent to that of cohorts receiving antifungal agents, within the same set of experiments.

### 3.3. Antifungal Bioassay

#### 3.3.1. Agar Plate Bioassay: Filamentous Fungi

In the plate bioassay, measurement of sensitivities of filamentous fungi to the antifungal agents was based on percent (%) radial growth of treated compared to control (“No treatment”) fungal colonies (See text for test concentrations.) [38]. Minimum inhibitory concentration (MIC) values on agar plates were determined based on triplicate bioassays, and defined as the lowest concentration of agents where no fungal growth was visible on the plate. For the above assays, fungal conidia (5 × 10^4^ CFU/mL) were diluted in phosphate-buffered saline (PBS) and applied as a drop onto the center of PDA plates with or without antifungal compounds. Growth was observed for three to seven days to determine cellular sensitivities to drugs/compounds.

#### 3.3.2. Microtiter Plate (microdilution) Bioassay: Filamentous Fungi

To determine antifungal chemosensitizing activities of KA (0.2, 0.4, 0.8, 1.6, 3.2, 6.4, 12.8 mM) to antifungal drug (AMB; 0.125, 0.25, 0.5, 1, 2, 4, 8, 16, 32 μg/mL) or H_2_O_2_ (0.0625, 0.125, 0.25, 0.5, 1, 2, 4, 8, 16 mM) in filamentous fungi, checkerboard bioassays (0.4 × 10^4^–5 × 10^4^ CFU/mL) were performed in microtiter wells using a broth microdilution (with RPMI 1640 medium; Sigma Co. (St. Louis, MO, USA), according to methods outlined by the Clinical Laboratory Standards Institute (CLSI) M38-A [20]. MICs for chemosensitization were defined as the concentrations where no fungal growth was visible at 48 and 72 h. All bioassays were performed in triplicate. Statistical analysis was based on [22].

#### 3.3.3. Microtiter Plate (microdilution) Bioassay: Yeasts

Chemosensitizing activities of KA (0.2, 0.4, 0.8, 1.6, 3.2, 6.4, 12.8 mM) to antifungal drug (AMB; 0.125, 0.25, 0.5, 1, 2, 4, 8, 16, 32 μg/mL) or H_2_O_2_ (0.0625, 0.125, 0.25, 0.5, 1, 2, 4, 8, 16 mM) were determined by using checkerboard bioassays in microtiter plates (with RPMI 1640 medium, except SG for *S. cerevisiae*; Sigma Co., Madrid, Spain). To determine changes in MICs of antifungal agents (*i.e*., drugs and chemosensitizers) in microtiter wells, checkerboard bioassays (0.5 × 10^5^ to 2.5 × 10^5^ CFU/mL) were performed using broth microdilution protocols according to methods outlined by the European Committee on Antimicrobial Susceptibility Testing (EUCAST) [29]. MICs for chemosensitization were defined as the concentrations where no fungal growth was visible at 24 and 48 h. All bioassays were performed in triplicate. Statistical analysis was based on [22].

## 4. Conclusions

In summary, enhancing antifungal interactions of KA in combination with H_2_O_2_, AMB, FLUD or Kre-Me were, as follows: (1) All *A. fumigatus* strains were sensitive to either KA + H_2_O_2_ or KA + AMB; (2) *A. terreus* strains were only sensitive to KA + H_2_O_2_; (3) *C. albicans* CAN276, *C. krusei* ATCC6258, *C. neoformans* CN24, *S. cerevisiae* were only sensitive to KA + AMB; and (4) *A. flavus* 3357, *A. parasiticus* 5862, *C. albicans* 90028, *C. krusei* CAN75, *C. tropicalis* CAN286 were marginally or not sensitive to any co-treatments; (5) *A. fumigatus* AF293 was more sensitive than the MAPK mutant strains to KA + Kre-Me. Thus, the antifungal chemosensitizing capacity of KA appears to be antifungal agent and/or fungal strain-specific. In conclusion, KA, a safe natural compound, may have a new use as an enhancer of certain commercial antifungal agents, such as AMB, H_2_O_2_ or strobilurin, against defined fungal pathogens. The enhancing effect appears to involve the modulation of the function of oxidative stress response system in the fungus. Further studies are warranted to determine the precise mechanism of action of KA for antifungal chemosensitization.

## Figures and Tables

**Table 1:** Fungal strains used in this study.

Fungal strains	Strain characteristics	Source/Reference
***Filamentous fungi***		

*Aspergillus flavus* 3357	Kojic acid producer, Human pathogen (aspergillosis), Plant pathogen	NRRL a
*A. parasiticus* 5862	Kojic acid producer, Plant pathogen	NRRL a
*A. fumigatus* AF293	Human pathogen (aspergillosis), Reference clinical strain	[17]
*A. fumigatus sakA*Δ	Human pathogen (aspergillosis), MAPK mutant derived from AF293	[17]
*A. fumigatus mpkC*Δ	Human pathogen (aspergillosis), MAPK mutant derived from AF293	[18]
*A. terreus* UAB673	Human pathogen (aspergillosis), Clinical isolate	CDC b
*A. terreus* UAB680	Human pathogen (aspergillosis), Clinical isolate	CDC b
*A. terreus* UAB698	Human pathogen (aspergillosis), Clinical isolate	CDC b

***Yeasts***		

*Candida albicans* 90028	Human pathogen (candidiasis), Reference clinical strain	ATCC c
*C. albicans* CAN276	Human pathogen (candidiasis), Clinical isolate	IHMT d
*C. krusei* 6258	Human pathogen (candidiasis), Reference clinical strain	ATCC c
*C. krusei* CAN75	Human pathogen (candidiasis), Clinical isolate	IHMT d
*C. tropicalis* CAN286	Human pathogen (candidiasis), Clinical isolate	IHMT d
*Cryptococcus neoformans* CN24	Human pathogen (cryptococcosis), Clinical isolate	IHMT d
*Saccharomyces cerevisiae* BY4741	Model yeast, Parental strain (*Mat* a *his3*Δ*1 leu2*Δ*0 met15*Δ*0 ura3*Δ*0*)	SGD e
*S. cerevisiae bck1*Δ	MAPK mutant derived from BY4741	SGD e
*S. cerevisiae slt2*Δ	MAPK kinase kinase mutant derived from BY4741	SGD e

a NRRL, National Center for Agricultural Utilization and Research, USDA-ARS, Peoria, IL, USA.

b CDC, Centers for Disease Control and Prevention, Atlanta, GA, USA.

c ATCC, American Type Culture Collection, Manassas, VA, USA.

d IHMT, Instituto de Higiene e Medicina Tropical/CREM, Universidade Nova de Lisboa, Portugal.

e SGD, *Saccharomyces* Genome Database [19].

**Table 2:** Antifungal chemosensitization of kojic acid (mM) with H_2_O_2_ (mM) tested against *Aspergillus* strains. a Minimum fungicidal concentrations (MFCs) are concentrations where ≥99.9% fungal death was achieved.

Strains	Compounds	MIC alone	MIC combined	FICI	MFC alone	MFC combined	FFCI
*A. fumigatus*	Kojic	>12.8 b	0.8	**0.5**	>12.8	0.8	**0.5**
AF293	H_2_O_2_	8	4		8	4	
*A. fumigatus*	Kojic	>12.8	12.8	1.0	>12.8	12.8	1.0
*sakA*Δ	H_2_O_2_	4	2		4	2	
*A. fumigatus*	Kojic	>12.8	12.8	1.0	>12.8	12.8	1.0
*mpkC*Δ	H_2_O_2_	4	2		4	2	
*A. terreus*	Kojic	>12.8	6.4	0.8	>12.8	12.8	0.8
UAB673	H_2_O_2_	2	1		4	1	
*A. terreus*	Kojic	>12.8	6.4	0.8	>12.8	12.8	1.0
UAB680	H_2_O_2_	2	1		2	1	
*A. terreus*	Kojic	>12.8	6.4	0.8	>12.8	12.8	1.0
UAB698	H_2_O_2_	2	1		2	1	

Mean	Kojic	>12.8	7.6	0.8	>12.8	10.8	0.9
	H_2_O_2_	3.7	1.8		4.0	1.8	

*t*-test	Kojic	-	*p* < 0.001	-	-	*p* < 0.001	-
	H_2_O_2_		*p* < 0.5			*p* < 0.1	

a MIC: Minimum inhibitory concentration, MFC: Minimum fungicidal concentration, FICI: Fractional Inhibitory Concentration Indices, FFCI: Fractional Fungicidal Concentration Indices. Student’s *t*-test for paired data (combined, *i.e*., chemosensitization) was *vs*. mean MIC or MFC of each compound (alone, *i.e*., no chemosensitization) determined in six strains. Calculation was based on [22].

b Kojic acid was tested up to 12.8 mM. For calculation purpose, 25.6 mM (doubling of 12.8 mM) was used.

**Table 3:** Antifungal chemosensitization of kojic acid (mM) with AMB (μg/mL) tested against *Aspergillus* and yeast strains. a MFCs are concentrations where ≥99.9% fungal death was achieved, except where noted in the Table.

Strains	Compounds	MIC alone	MIC combined	FICI	MFC alone	MFC combined	FFCI
*A. fumigatus*	Kojic	>12.8 b	0.8	0.8	>12.8	3.2	0.6
AF293	AMB	4	2		>32 c	32	(99.8% inhibition)
*A. fumigatus*	Kojic	>12.8	12.8	1.0	>12.8	0.4	**0.5**
*sakA*Δ	AMB	2	1	(85%–90% inhibition)	4	2	
*A. fumigatus*	Kojic	>12.8	0.2	**0.5**	>12.8	0.2	**0.5**
*mpkC*Δ	AMB	4	2		8	4	
*C. albicans*	Kojic	>12.8	6.4	0.8	>12.8	> 12.8	2.0
CAN276	AMB	1	0.5		1	1	
*C. krusei*	Kojic	>12.8	0.4	**0.5**	>12.8	6.4	0.8
ATCC 6258	AMB	2	1		2	1	
*Cryptococcus*	Kojic	>12.8	0.4	**0.5**	>12.8	3.2	0.6
*neoformans* CN24	AMB	2	1		2	1	

Mean	Kojic	>12.8	3.5	0.7	>12.8	6.5	0.8
	AMB	2.5	1.3		13.5	6.8	

*t*-test	Kojic	-	*p* < 0.001	-	-	*p* < 0.005	-
	AMB		*p* < 0.05			*p* < 1.0	

a AMB: Amphotericin B. MIC: Minimum inhibitory concentration, MFC: Minimum fungicidal concentration. FICI: Fractional Inhibitory Concentration Indices, FFCI: Fractional Fungicidal Concentration Indices. Student’s *t*-test for paired data (combined, *i.e*., chemosensitization) was *vs*. mean MIC or MFC of each compound (alone, *i.e*., no chemosensitization) determined in six strains. Calculation was based on [22].

b Kojic acid was tested up to 12.8 mM. For calculation purpose, 25.6 mM (doubling of 12.8 mM) was used.

c AMB was tested up to 32 μg/mL. For calculation purpose, 64 μg/mL (doubling of 32 μg/mL) was used.

**Table 4:** Antifungal chemosensitization of kojic acid (mM) with AMB (μg/mL). a MFCs are concentrations where ≥99.9% fungal death was achieved.

Strains	Compounds	MIC alone	MIC combined	FICI	MFC alone	MFC combined	FFCI
*S. cerevisiae*	Kojic	>12.8 b	6.4	0.8	> 12.8	12.8	1.0
BY4741	AMB	2	1		4	2	
*S. cerevisiae*	Kojic	>12.8	6.4	0.8	> 12.8	12.8	1.0
*slt2*Δ	AMB	2	1		4	2	
*S. cerevisiae*	Kojic	>12.8	6.4	0.8	> 12.8	12.8	1.0
*bck1*Δ	AMB	2	1		4	2	

Mean	Kojic	>12.8	6.4	0.8	> 12.8	12.8	1.0
	AMB	2	1		4	2	

*t*-test	Kojic	-	*p* < 0.001	-	-	*p* < 0.001	-
	AMB		*p* < 0.001			*p* < 0.001	

a AMB: Amphotericin B. MIC: Minimum inhibitory concentration, MFC: Minimum fungicidal concentration. FICI: Fractional Inhibitory Concentration Indices, FFCI: Fractional Fungicidal Concentration Indices. Student’s *t*-test for paired data (combined, *i.e*., chemosensitization) was *vs*. mean MIC or MFC of each compound (alone, *i.e*., no chemosensitization) determined in three strains. Calculation was based on [22].

b Kojic acid was tested up to 12.8 mM. For calculation purpose, 25.6 mM (doubling of 12.8 mM) was used.

**Table 5:** Summary of responses of *Aspergillus* and yeast strains to the co-application of kojic acid with H_2_O_2_ or AMB. a

Fungal strains	Agents co-applied	

	H_2_O_2_ (FICI, FFCI) b	AMB (FICI, FFCI) b
***Filamentous fungi***		

*Aspergillus flavus* 3357	-	-
*A. parasiticus* 5862	-	-
*A. fumigatus* AF293	+ (**0.5**, **0.5**)	+ (0.8, 0.6)
*A. fumigatus sakA*Δ	+ (1.0, 1.0)	+ (1.0, **0.5**)
*A. fumigatus mpkC*Δ	+ (1.0, 1.0)	+ (**0.5**, **0.5**)
*A. terreus* UAB673	+ (0.8, 0.8)	-
*A. terreus* UAB680	+ (0.8, 1.0)	-
*A. terreus* UAB698	+ (0.8, 1.0)	-

***Yeasts***		

*Candida albicans* 90028	-	-
*C. albicans* CAN276	-	+ (0.8, 2.0)
*C. krusei* 6258	-	+ (**0.5**, 0.8)
*C. krusei* CAN75	-	-
*C. tropicalis* CAN286	-	-
*Cryptococcus neoformans* CN24	-	+ (**0.5**, 0.6)
*Saccharomyces cerevisiae* BY4741	-	+ (0.8, 1.0)
*S. cerevisiae bck1*Δ	-	+ (0.8, 1.0)
*S. cerevisiae slt2*Δ	-	+ (0.8, 1.0)

a +, enhancement of antifungal activity after co-application; -, no enhancement of antifungal activity after co-application.

b FICI, Fractional Inhibitory Concentration Indices; FFCI, Fractional Fungicidal Concentration Indices; Both FICI and FFCI values were based on Tables 2–4; Bold: synergistic interaction.
